# Transcultural adaptation and new proposal for the nursing outcome, ****Physical condition**** (2004)

**DOI:** 10.1590/1518-8345.2412.2984

**Published:** 2018-05-17

**Authors:** Jessica Rojas Navarrete, Paloma Echevarría Pérez, César Leal Costa

**Affiliations:** 1 Msc, Professor, Universidad Católica San Antonio de Murcia, Murcia, Spain.; 2 PhD, Professor, Universidad Católica San Antonio de Murcia, Murcia, Spain.; 3 PhD, Professor, Universidad Católica San Antonio de Murcia, Murcia, Spain.

**Keywords:** Physical Fitness, Nursing, Nursing Evaluation, Nursing Outcomes, Validation Studies

## Abstract

**Objectives:**

cross-culturally adapt to the Spanish context and make a new proposal for the nursing outcome, *Physical Condition (2004),* of the Nursing Outcomes Classification (NOC) for its precise use in clinical practice.

**Method:**

a cross-cultural adaptation study and a proposal for the nursing outcome, *Physical Condition,* was conducted and supported by the opinion of 26 experts. The data was obtained through an electronic form, and a quantitative analysis was conducted, using the SPSS software.

**Results:**

the version adapted to the Spanish context was obtained and the proposal of the outcome, *Physical Condition,* received agreement from 26 experts, with a mean score greater than 7.6 for adequacy of the outcome definition and its indicators, and 8.5 for the relevance of the indicators.

**Conclusions:**

the version adapted to the Spanish context and a new proposal for *Physical Condition* were obtained. The results obtained indicate a high level of adequacy and relevance, an instrument of great utility in the clinic, and research was obtained to evaluate the interventions directed to the improvement of the physical condition.

## Introduction

Studies show that the problem of physical inactivity is a global concern, and indicate that a large number of individuals in the population do not follow the recommendations on the practice of physical activity^1^. The latest available data indicate that, on a global level, approximately 23% of adults, aged 18 or older, do not meet the minimum recommendations for physical activity (20% of men and 27% of women), with proportions ranging from 15% in Southeast Asia to around 36% in the American continent and the Eastern Mediterranean. These figures show that, on an international level, one out of every four adults is not sufficiently active, which represents more than 80% of the adolescent population, with an age ranging from 11 to 17 years old^2^ ^-^ ^3^. In the European Union, the prevalence of sedentary lifestyle is high^4^.

The physical condition, or physical form, is a set of qualities that allows people to carry out their activities of daily life with vigor and caution, without excessive fatigue, and with enough energy to enjoy leisure activities and face unforeseen emergencies^5^. The level of physical condition that a person possesses is a significant indicator of health risks. This fact highlights the importance of preventive medicine’s recommendation for increasing the performance of physical activity, and the need to have an accurate, simple, and cost-effective measurement instrument to assess the level of physical condition^6^.

The concept of physical condition has evolved historically. Thus a concept called *Physical Condition Related to Sports Performance* ^7^ has been differentiated, and another concept has been linked to a biomedical approach: *Physical Condition Related to Health* ^8^. The concept, *Physical Condition Related to Health,* encompasses those components of physical condition that are linked to the health status of a person, and that may be determined by the performance of physical activity on a regular basis^9^. This concept is defined as the state of physical and physiological characteristics that indicate the existence of premature risk of developing certain diseases or morbidity, which is influenced by a sedentary lifestyle^10^.

Considering this, nurses, who work first hand with the population that suffers the effects of an increase in sedentary lifestyle^11^, could help users of the different healthcare units improve their health status, including the evaluation of their physical condition^12^. Moreover, the evaluation of the components of the physical condition related to health, and the linking of the outcomes with other health measures, would allow nurses to theoretically document the effects produced by performance of physical exercise, or the lack thereof.

Finally, we considered that nurses rarely use objective measures to evaluate the P*hysical Condition Related to Health,* ^13^ and that although the NOC was designed to measure outcomes sensitive to nursing practice, its sensitivity has not yet been sufficiently studied^14^ and it has been minimally explored in the clinical setting. Therefore, we consider the transcultural adaptation of the measuring instrument for the nursing outcome, *Physical Condition (2004),* and obtaining empirical evidence of content validity and consensus of the proposed outcome, *Physical Condition Related to Health*, to be important because it is determinant that an instrument adapted cross-culturally is adequately developed and validated through the analysis of satisfactory psychometric properties^15^. The existing nursing outcome, *Physical condition (2004),* is linked to the health field and is composed of 13 indicators related to the health status of a person that can be assessed with a 5-point Likert scale, in which a «5» is the best possible score and a «1» is the worst possible score^16^.

The objectives of this study were to cross-culturally adapt the nursing outcome, *Physical Condition* *(2004),* of the 5th edition of the NOC, to the Spanish context and to make a consensus proposal by expert opinion of the nursing outcome, *Physical Condition Related to Health.*

## Method

The study that was conducted was a cross-cultural adaptation of the nursing outcome, *Physical Condition,* and of its indicators, from the NOC classification, 5th edition ^17^ and a new proposal of said outcome, based on the opinion of experts between January of 2015 and September of 2016.

Thus, to achieve the objectives, the study was conducted in two different phases:

Phase 1. - Adaptation of the nursing outcome, *Physical Condition,* of the 5th Edition NOC, to the Spanish context.

The transcultural adaptation of the original version to the Spanish context was conducted according the process described by Beaton et al.^18^ which consists of the following stages:

1) Initial translation: first, two translations of the nursing outcome, *Physical Condition,* were performed from the source language (English) to the target language (Spanish), obtaining two versions in Spanish, named respectively, translation 1 (T1) and translation 2 (T2). In this first stage, the researchers and the translators compared both translations, and the discrepancies that arose were agreed upon. The translations were performed by two bilingual translators whose mother tongue was Spanish, and who had different professional profiles. Translator 1 was a health sciences and physical education professional, specifically, a graduate in psychology and a professional athlete with knowledge about the concepts that the measuring instrument evaluates. Translator 2 was a graduate professional in English studies, who had no knowledge of the concepts of the instrument, and was not related to health sciences or physical activity. 2) Synthesis of the translations: in this stage, starting from the two translations into Spanish (T1 and T2) and the original version, we made a synthesis of these translations to obtain a common version called translation 12 (T12). 3) Back translation: starting from the T12 version, and without knowing or seeing the original version, two translators translated the instrument into the original language, to make sure that the translated version reflected the same indicators contained in the original version, resulting in two versions in English, called back-translation 1 (RT1) and back-translation 2 (RT2). In the third stage, the two translators that produced the back translations, RT1 and RT2, were two people whose mother tongue was English and who did not have knowledge about the concepts explored in the measuring instrument, nor were they trained in the health sciences or in physical activity; in particular, they were graduates in English philology who worked as teachers and researchers at the Catholic University of San Antonio de Murcia (Spain). 4) Expert Panel: At this stage, a panel of experts was created, consisting of all the translators who had prepared the different translations. The group of experts consolidated all the versions of the measuring instrument, the original version and each of the different translations (T1, T2, RT1 and RT2), with their corresponding reports on the contributions of the translators, creating a final consensus version in Spanish.

Phase 2 - New proposal of the revised version of the instrument for measuring the nursing outcome, *Physical Condition,* of the NOC 5th edition, based on the opinion of experts.

The second phase of the investigation was composed of the stages described below. 1) Quantitative analysis of the definition of *Physical Condition Related to Health,* through the opinion of experts, to obtain theoretical evidence of face, consensus and content validity. After the bibliographic search in different databases (PubMed, Cochrane, Ebsco, ISI Web of Knowledge and Teseo) and the creation of a proposal of the definition of the nursing outcome, *Physical Condition Related to Health,* and of the main components detected in the literature: cardiorespiratory capacity, musculoskeletal capacity, weight and body composition, and motor capacity^19^, a quantitative analysis was conducted through a consultation with a group of experts. The group of experts was comprised of professionals who met the following inclusion criteria: graduates in a discipline such as medicine, nursing, physiotherapy, or physical activity and sport sciences; professional experience in teaching, research or another health care field for at least two years; scientific academic production in the field of physical exercise and health, or nursing taxonomies. The exclusion criterion was not meeting all the inclusion criteria. The experts collaborated voluntarily. The type of sampling five to ten experts from each professional group referred to above^20^. Specifically, we had a sample of 26 experts. For the quantitative analysis of the definition of the proposed nursing outcome, an online form was sent to the email address of the selected experts that contained the proposed definition of said outcome, *Physical Condition Related to Health*. The purpose of the form was explained to them in the e-mail, and they were asked to participate voluntarily in the research study, as well as provided instructions for correct completion. The form was developed through the electronic application, Google Forms. In it, the experts had to evaluate, on a scale of 1 to 10, the suitability of the proposed definition, where 1 = not at all adequate, and 10 = totally adequate. Likewise, the experts also had to assess the relevance of the different proposed indicators of the outcome, *Physical Condition Related to Health*, with a scale from 1 to 10, where 1 = not relevant and 10 = very relevant, and the suitability of the definition proposed for each indicator. They were also allowed to express, through an open question, any other personal contribution on the definition of the outcome, and the relevance or suitability of the proposed indicators. On the other hand, in the last sections of the form, a question was included asking the experts to indicate which indicator they considered as the gold standard among those proposed, if they considered it appropriate, and what others indicators of the *Physical Condition Related to Health* were considered relevant but not included in the proposed definition. The statistical analysis performed for each definition was a calculation of the arithmetic mean of the *adequacy* variable. Thus, we were able to measure the level of adequacy that the experts assigned to each definition, in which the higher the score obtained, the better adequacy, and vice versa. Likewise, the limits of the distribution and variance were calculated to measure the degree of consensus; high variance scores indicate a greater degree of discrepancy over the adequacy of the definitions, while low scores indicate a lower degree of discrepancy. For the *relevance* variable, the mean, variance, limits of the distribution, frequency, and percentage were also calculated, according to the scores assigned by the experts to each proposed indicator. 2) Proposal of the revised version of the instrument for measuring the nursing outcome, *Physical Condition,* of the NOC 5th edition: according to the scores obtained from the group of experts that showed the high level of adequacy and relevance, and starting from the consensus of the definition and of the indicators of the outcome under study, we proceeded to establish the indicators of the proposed nursing outcome, called *Physical Condition* *related to health*, as the gold standard indicator, carrying out a new proposal for the revised version of the nursing outcome, *Physical Condition* of the NOC 5th edition.

The software used for the statistical analysis of the data of both variables was SPSS, version 24.

### Ethical aspects

The present study was approved by the Clinical Research Ethics Committee of the Hospital Clínico Universitario Virgen de la Arrixaca, belonging to Health Area 1 (Murcia-West).

## Results

Phase 1. - Adaptation of the nursing outcome, *Physical Condition,* of the NOC 5th edition, to the Spanish context.

**Figure 1:**
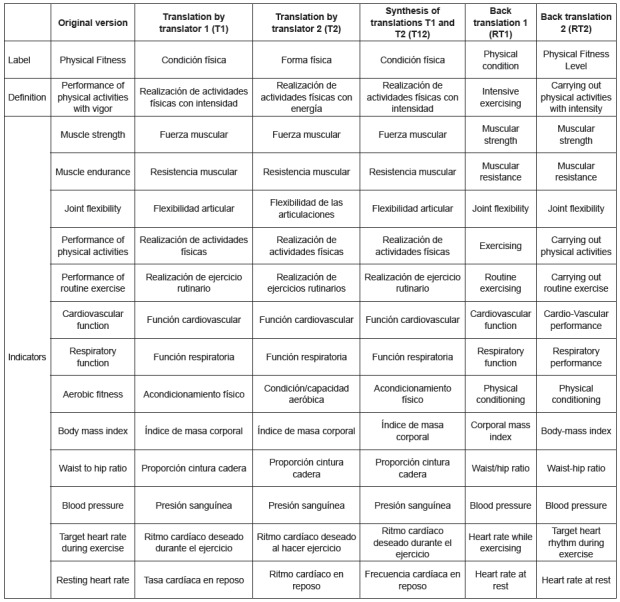
Synthesized version of the T1 and T2 translations to the Spanish context and back translations T1 and T2 of the nursing outcome, *Physical* *Condition,* of the NOC 5th edition, by the group of experts, Murcia, Spain, 2015, 2016

**Figure 2:**
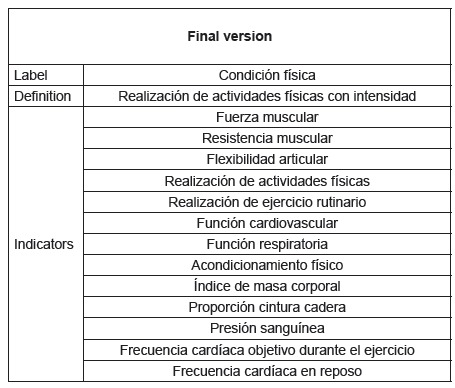
Final consolidated version of the nursing outcome, *Physical Condition,* of the NOC 5th edition, by the group of experts, based on the original version and the translated versions (T1, T2, T12, RT1 and RT2). Murcia, Spain, 2015, 2016

The results obtained in the first phase of the study, that is, the cross-cultural adaptation to the Spanish context of the nursing outcome, *Physical Condition* of the NOC 5th edition, show the translated versions of said outcome that are detailed in Figure 1 and Figure 2.

Phase 2. - New proposal of the revised version of the instrument for measuring the nursing outcome, *Physical Condition,* of the NOC 5th edition, based on expert opinion.

In the second phase of the study, 26 forms were obtained, duly completed, with the opinion of the selected experts on the definition of the proposed nursing outcome, *Physical Condition Related to Health*. The origin of the experts who participated in the round of work shows the diversity of health and physical activity professionals, who teach, conduct research or provide direct care in various centers and services of sports medicine, Chair in sports, and faculties of health sciences (nursing, physiotherapy, physical activity and sports sciences), who contributed their knowledge to this study. Likewise, the largest number of experts came from the nursing faculty at the Facultad de Enfermería de la Universidad Católica San Antonio de Murcia, as presented in Table 1.

**Table 1:** Frequency and percentage of the distribution of experts according to the university, research group or work center to which they belong. Murcia, Spain, 2015, 2016.

Spanish Universities and Research Groups	n*	%^†^
Centro Regional de Medicina del Deporte de Valladolid	2	7.7
Cátedra de Fisiología del Deporte de la UCAM^‡^	2	7.7
Cátedra de Traumatología del Deporte de la UCAM^‡^	3	11.5
Centro de Alto Rendimiento del San Cugat del Vallés	3	11.5
Cátedra Internacional de Ecografía Músculo Esquelética de la UCAM^‡^	1	3.8
Cátedra Internacional de Medicina del Deporte de la UCAM^‡^	1	3.8
Facultad de Ciencias de la Actividad Física y del Deporte de la UCAM^‡^	1	3.8
Facultad de Ciencias de la Actividad Física y del Deporte de la Universidad de Murcia	1	3.8
Facultad de Enfermería de la UCAM^‡^	5	19.2
Facultad de Enfermería de la Universidad Cardenal Herrera	1	3.8
Facultad de Enfermería de la Universidad de Valencia	1	3.8
Facultad de Ciencias de la Salud de la UCAM^‡^ (Grado en Fisioterapia)	2	7.7
Escuela del Deporte y la Salud Mediterráneo Activo de Málaga	1	3.8
Servicio de Cardiología Hospital Universitario Virgen de La Arrixaca	1	3.8
Escuela de Medicina del Deporte de la Universidad de Oviedo	1	3.8

*n = 26 subjects; ^†^% = percentage; ^‡^UCAM = Universidad Católica San Antonio de Murcia

The statistical analysis of the data showed that the scores obtained after consultation with the experts revealed mean scores above 7.6 in the adequacy of the definition of the proposed nursing outcome, *Physical Condition Related to Health,* as well as in the indicators raised, representing a high level of adequacy. However, although there were differences between the assigned scores, the variance had low values, as can be seen in Table 2. Likewise, 65.39% and 73.07% of the experts assigned scores between 8 and 10 for adequacy of the definition and of the proposed indicators, respectively.

**Table 2:** Descriptive results of the scores obtained in the adequacy and relevance of the semantic definition and the indicators of the proposed nursing outcome, Physical condition related to health, after consulting the experts in physical condition and health, Murcia, Spain, 2015, 2016.

Outcome and indicators	Adequacy			Relevance	
	Limits	Mean	Variance	Limits	Mean
Outcome Physical condition related to health	3-10	7.62	4.09	-	-
Indicator Cardiorespiratory capacity	2-10	8.15	3.36	5-10	8.81
Indicators Muscular strength Flexibility	1-10	7.62	6.14	7-10	8.81
Indicators Body mass index Waist circumference	2-10	7.85	3.89	3-10	8.23
Indicator Balance	1-10	7.85	4.70	5-10	8.15

N=26

In relation to the statistical analysis of the scores obtained for the relevance of the indicators of the proposed outcome, the average scores were above 8.15, which indicates high relevance, and the variance was low, revealing low discrepancy between the contributions of the experts (Table 2). A majority, 81.73%, of the experts assigned scores between 8 and 10 on the relevance of the proposed indicators.

As for other indicators suggested by the group of experts for *Physical Condition Related to Health*, the results obtained suggested the inclusion of these indicators: *muscle elasticity*, *fat-free mass*, *mobility or range of motion*, *body fat* *percentage,* and *speed of reaction*, although all of them had a very low percentage (3.85% -11.54%). However, *body fat* *percentage* was the indicator that had the highest score (11.54%) and was included in the proposal, due to its significance in the assessment of physical condition in the health field^21^.

Regarding the star indicator raised by the group of experts, the *cardiorespiratory capacity* indicator was the one with the highest assigned score (34.62%), therefore, since it is an essential component of *Physical Condition Related to Health* ^22^, we consider that this indicator is a priority in the study of the construct object of the present investigation.

**Figure 3:**
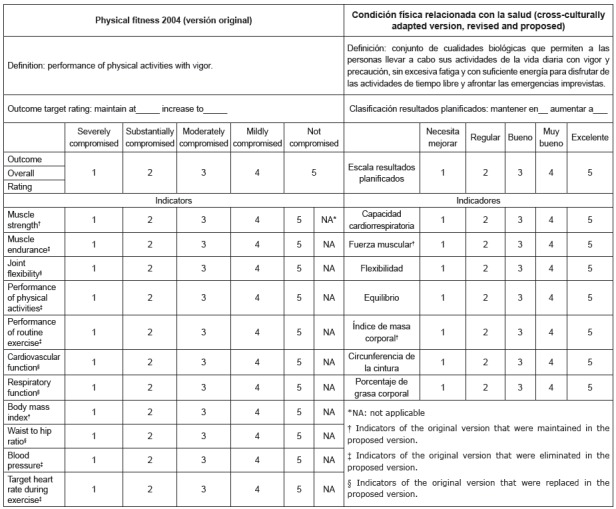
Original and cross-culturally adapted versions, revision, and proposal of the instrument for measuring the nursing outcome, *Physical* *Condition,* and its indicators of the NOC, 5th edition. Murcia, Spain, 2015, 2016

As a final result of the study, the proposal of the nursing outcome, *Physical Condition Related to Health,* was developed and agreed to by 26 experts, comprised of seven indicators; this proposal is presented next to the original version, in Figure 3.

## Discussion

The use of a measuring instrument in a clinical environment, different from the one of origin, requires a process of cultural adaptation to verify semantic equivalence^23^. The first two versions of English to Spanish of the nursing outcome, *Physical Condition,* (T1 and T2) by the selected translators facilitated the conceptual, cultural, idiomatic, and semantic adaptation in the Spanish context by these translators, resulting in a final synthesized version of English to Spanish (T12). The following phases of *back translation* and *synthesis of the back translations* contributed to the verification of the suitability of the T12 version, thus allowing us to initiate the second stage of the investigation.

In the proposal of the nursing outcome, *Physical Condition,* of the 5th edition NOC classification, adapted to the Spanish context, and agreed upon by consulting experts, the definition of the outcome, *Physical Condition Related to Health,* had a high level of adequacy for which said definition was proposed for the revised nursing outcome, since the physical condition not only implies carrying out physical activities with vigor^17^, but also reflects the ability to perform the basic activities of daily life and maintain good health^9^. *Expert judgment* is a fundamental procedure that would be framed within the content validity^24^. In addition to content validity, evidence of consensus validity is obtained that is obtained by feedback from the experts, and achieving agreement on the contents of the instrument.

The changes made to the label, definition and indicators of the proposed version, with respect to the original version of the 5th Edition NOC, in English, indicate that the original nursing outcome required revision for its precise use in the clinical setting, and in the Spanish context.

Regarding the label of the nursing outcome, *Physical Condition*, a modification has been made by introducing a more specific label to the area of interest and study, that is, *Physical Condition Related to Health*. The concept of physical condition related to health encompasses those components of physical condition that are linked to the health status of a person, and that may be determined by the performance of physical activity on a regular basis^10^.

In reference to the definition of the nursing outcome, *Physical Condition,* of the 5th edition NOC, this did not cover the totality of the set of aspects referred to by the concept of *Physical Condition Related to Health,* as it is a very brief and incomplete definition. After consultation with the experts, the definition of the proposed version of the outcome, *Physical Condition Related to Health,* was agreed upon and developed to obtain a Spanish version adapted semantically and culturally from the revised nursing outcome: *a set of biological qualities that allow people to carry out their daily life activities with vigor and caution, without excessive fatigue and with enough energy to enjoy leisure activities and face unforeseen emergencies*.

Mentioning the indicators of the nursing outcome studied, and according to the opinion of the experts consulted, since it is considered that the evaluation process conducted by a committee of experts on the content of the items demonstrates content validity of the adapted instrument for the other culture ^25^ ^-^ ^26^, two indicators of the original version have been preserved: *muscle strength* and *body mass index*, as they are all components of the physical condition in the health field^27^. On the other hand, six indicators existing in the original version were eliminated: *performance of physical activities, performance of routine exercise, physical conditioning, blood pressure, target heart rate during exercise, and resting heart rate,* since, although some authors^10^ consider that the blood pressure indicator is a component of the physical condition related to health, it is not considered as a main component, as well as the other components ^9^ ^,^ ^19^. Likewise, the components, *cardiovascular function* and *respiratory function,* were replaced by *cardiorespiratory capacity* component, *waist-hip ratio* was replaced by *waist circumference,* and *joint flexibility* was replaced by *flexibility*. Finally, we have included 2 new indicators that are *balance* and *body* *fat percentage*, since they are essential dimensions of the physical condition related to health ^21^ ^-^ ^22^.

The knowledge of the adult population’s state of physical condition is an important indicator of health, and there is currently a deficit of instruments and studies written in Spanish that assess it^28^. Physical condition is an independent marker for cardiovascular disease risk^29^, although it can be immensely influenced by lifestyle^30^.

Currently there are few instruments that allow us to evaluate physical condition in a simple, economic, and accurate way^28^. Several authors have described easy-to-use instruments that do not require the use of highly sophisticated technological equipment, and that have been validated by questionnaires^31^ ^-^ ^32^. However, due to the importance of determining the status of this important health indicator, the transcultural adaptation of the measurement instrument of the nursing outcome, *Physical Condition* (2004), to the Spanish context and the proposal of the outcome, *Physical Condition Related to Health*, validated conceptually, through the opinion of a group of experts, it is a great contribution for its safe use in clinical practice, in the Spanish context.

The content validation evaluation analyzes the representativeness or adequacy of the content of the measuring instrument^25^, what constitutes a necessary initial stage, which is of great importance in the proposal made for the nursing outcome, Physical Condition Related to Health; this allows the understandable application of the instrument to Spanish^33^. However, in order to be used by nurses working in the healthcare field, it is necessary to conclude the clinical validation process, analyzing all the metric properties of said measuring instrument in a large sample.

## Conclusions

The study updated the existing knowledge on the nursing outcome, *Physical Condition (2004),* of the 5th Edition NOC when being adapted cross-culturally from the original English version, to the Spanish context, obtaining a version in Spanish language with the same semantic equivalences. It is considered that the conceptual validation of nursing outcomes is highly relevant for the development of standardized nursing language, since it contributes to the improvement of communication among nursing professionals, the application and documentation of the nursing process and the advancement of nursing knowledge.

The proposed nursing outcome, *Physical Condition Related to Health,* reflects the state of health studied in a more precise way, which will be able to facilitate the systematized planning and implementation of nursing care for nurses. However, the importance of continuing the analysis of the rest of the psychometric properties, in future studies, is highlighted.

## References

[1] Guedes NG, Lopes MV, Moreira RP, Cavalcante TF, de Araujo TL (2010). Prevalence of sedentary lifestyle in individuals with high blood
pressure. Int J Nurs Knowl.

[2] World Health Organization (2017). Physical Inactivity: A Global Public Health Problem.

[3] World Health Organization (2017). Phys Inactivity.

[4] Guirao-Goris JA, Cabrero-García J, Moreno-Pina JP, Muñoz-Mendoza CL (2009). Structured review of physical activity measurement with
questionnaires and scales in older adults and the elderly. Gac Sanit.

[5] Malina RM, Bouchard C, Bar-Or O (2004). Growth, maduration, and physical activity.

[6] Heimmel J, Patel S, Cody R, Bachmann G (2007). Evaluation of physical fitness in an ambulatory
setting. Am J Obstet Gynecol.

[7] Pate RR (1988). The evolving definition of physical fitness. Quest.

[8] Júdice PB, Silva AM, Berria J, Petroski EL, Ekelund U, Sardinha LB (2017). Sedentary patterns, physical activity and health-related physical
fitness in youth a cross-sectional study. Int J Behav Nutr Phys Act.

[9] Barton M, Jackson AW, Martin SB, Morrow JR, Petrie TA, Greenleaf CA (2017). Better health-related fitness in youth: implications for public
health guidelines. Int J Exerc Sci.

[10] Bouchard C, Shepard RJ, Bouchard C, Shephard RJ, Stephens T (1994). Physical activity, fitness and health: The model and key
concepts. Physical activity, fitness and health: International proceedings and
consensus statement.

[11] Manson JE, Skerrett PJ, Greenland P, VanItallie TB (2004). The escalating pandemics of obesity and sedentary lifestyle A
call to action for clinicians. Arch Intern Med.

[12] Matheson GO (2004). Why settle for baseline health and fitness. Phys Sportsmed.

[13] Buchholz SW, Purath J (2007). Physical activity and physical fitness counseling patterns of
adult nurse practitioners. J Am Acad Nurse Pract.

[14] Schneider JS, Barkauskas V, Keenan G (2008). Evaluating home health care nursing outcomes with OASIS and
NOC. J Nurs Scholarsh.

[15] Pereira FMV, Lam SC, Gir E (2017). Cultural adaptation and reliability of the Compliance with
Ctandard Precautions Scale (CSPS) for nurses in Brazil Rev.
Latino-Am. Enfermagem.

[16] Moorhead S, Johnson M, Maas M, Swanson E (2013). Nursing Outcomes Classification (NOC)..

[17] Muñiz J, Elosua P, Hambleton RK (2013). Directrices para la traducción y adaptación de los test
2.ed. Psicothema.

[18] Beaton DE, Bombardier C, Guillemin F, Ferraz MB (2000). Guidelines for the process of cross-cultural adaptation of
self-report measures. Spine.

[19] Knapik JJ (2015). The importance of physical fitness for injury prevention: part
1. J Spec Oper Med.

[20] De Villiers MR, de Villiers PJ, Kent AP (2005). The Delphi technique in health sciences education
research. Med Teach.

[21] Ruiz JR, Castro-Piñero J, Artero EG, Ortega FB, Sjöström M, Suni J (2009). J Predictive validity of health-related fitness in youth: a
systematic review. Br J Sports Med.

[22] Ortega FB, Ruiz JR, Castillo MJ, Moreno LA, Urzanqui A, González-Gross M (2008). Health-related physical fitness according to chronological and
biological age in adolescents. The AVENA study. J Sports Med Phys Fitness.

[23] World Health Organization (2013). Process of translation and adaptation of instruments.

[24] Carretero-Dios H, Pérez C (2007). Normas para el desarrollo y revisión de estudios instrumentales:
consideraciones sobre la selección de test en la investigación
psicológica. Int J Clinic Health Psychol.

[25] Mokkink LB, Terwee CB, Knol DL, Stratford PW, Alonso J, Patrick DL (2010). The COSMIN checklist for evaluating the methodological quality of
studies on measurement properties: a clarification of its
content. BMC Med Res Methodol.

[26] Vituri DW, Matsuda LM (2009). Content validation of quality indicators for nursing care
evaluation. Rev Esc Enferm USP.

[27] Caspersen CJ, Powell KE, Christenson GM (1985). Physical activity, exercise, and physical fitness: definitions
and distinctions for health-related research. Public Health Rep.

[28] Ramírez-Velez R, Milena Cruz-Salazar S, Martínez M, Cadore EL, Alonso-Martínez AM, Correa-Bautista JE (2017). Construct validity and test-retest reliability of the
International Fitness Scale (IFIS) in Colombian children and adolescent aged
9-17 9 years: the FUPRECOL study. PeerJ.

[29] LaMonte MJ, Barlow CE, Jurca R, Kampert JB, Church TS, Lee D-C (2005). Cardiorespiratory fitness is inversely associated with the
incidence of metabolic síndrome a prospective study of men and
women. Circulation.

[30] Ortega FB, Ruiz JR, Castillo MJ, Sjöström M (2008). Physical fitness in childhood and adolescence: a powerful marker
of health. Int J Obes.

[31] Jackson AS, Sui X, O`Connor DP, Church TS, Lee D-C, Artero EG (2012). Longitudinal cardiorespiratory fitness algorithms for clinical
settings. Am J Prev Med.

[32] Ortega FB, Ruiz JR, España Romero V, Vicente-Rodríguez G, Martínez-Gómez D, Manios Y (2011). The International Fitness Scale (IFIS) usefulness of
self-reported fitness in youth. Int J Epidemiol.

[33] Freire MHS, Arreguy-Sena C, Müller PCS (2017). Cross-cultural adaptation and content and semantic validation of
the Difficult Intravenous Access Score for pediatric use in Brazil Rev.
Latino-Am. Enfermagem.

